# A novel method to prepare L-Arabinose from xylose mother liquor by yeast-mediated biopurification

**DOI:** 10.1186/1475-2859-10-43

**Published:** 2011-06-07

**Authors:** Hairong Cheng, Hengwei Wang, Jiyang Lv, Mingguo Jiang, Shuangjun Lin, Zixin Deng

**Affiliations:** 1 Laboratory of Microbial Metabolism and School of Life Sciences and Biotechnology, Shanghai Jiao Tong University, 800# Dongchuan Road, Shanghai, China; 2 School of Chemistry and Ecology Engineering, Guangxi University for Nationalities, Nanning, China

## Abstract

**Background:**

L-arabinose is an important intermediate for anti-virus drug synthesis and has also been used in food additives for diets-controlling in recent years. Commercial production of L-arabinose is a complex progress consisting of acid hydrolysis of gum arabic, followed by multiple procedures of purification, thus making high production cost. Therefore, there is a biotechnological and commercial interest in the development of new cost-effective and high-performance methods for obtaining high purity grade L-arabinose.

**Results:**

An alternative, economical method for purifying L-arabinose from xylose mother liquor was developed in this study. After screening 306 yeast strains, a strain of *Pichia anomala *Y161 was selected as it could effectively metabolize other sugars but not L-arabinose. Fermentation in a medium containing xylose mother liquor permitted enrichment of L-arabinose by a significant depletion of other sugars. Biochemical analysis of this yeast strain confirmed that its poor capacity for utilizing L-arabinose was due to low activities of the enzymes required for the metabolism of this sugar. Response surface methodology was employed for optimization the fermentation conditions in shake flask cultures. The optimum conditions were: 75 h fermentation time, at 32.5°C, in a medium containing 21% (v/v) xylose mother liquor. Under these conditions, the highest purity of L-arabinose reached was 86.1% of total sugar, facilitating recovery of white crystalline L-arabinose from the fermentation medium by simple methods.

**Conclusion:**

Yeast-mediated biopurification provides a dynamic method to prepare high purity of L-arabinose from the feedstock solution xylose mother liqour, with cost-effective and high-performance properties.

## Background

The sugar, L-arabinose is named after gum arabic from which it was first isolated. It is a five-carbon sugar and is widely found in nature as a component of biopolymers such as hemicellulose and pectin. L-arabinose is traditionally used in the flavour industry in Maillard reaction and in culture media. Recently, L-arabinose has been used in food additives and as an intermediate in drug synthesis [1,2]. The effects of L-arabinose on intestinal absorption of sucrose have been investigated. Physiological experiments have revealed that L-arabinose inhibits the sucrase activity of intestinal mucosa [3-5]. It also suppresses increase of blood glucose after sucrose loading in a dose-dependent manner, but shows no effect after starch loading in mice [5]. These observations have suggested the possibility of application of L-arabinose, mixed with small quantities of sucrose, in controlled diets such as those for weight-loss or for diabetics [6].

Commercial production of L-arabinose consists of an initial step of acid hydrolysis of gum arabic, followed by its purification through multiple procedures such as neutralization reaction, ion exchange and other chromatographic separations. Recently, Lim et al. (2011) reported a new preparation method for L-arabinose from arabinan by the combination of endo-and exo-arabinanases, which yielded 16 g L^-1 ^L-arabinose from 20 g l^-1 ^arabinan (80% yield) [7]. Earlier, Ahmed et al. (1999) reported a novel L-arabinose production method using *Mycobacterium smegmatis *to transform ribitol, first by oxidization to L-ribulose that is then isomerized into L-arabinose [8]. However, these methods need expensive raw materials and complex separation processes for purification of L-arabinose, which render these methods impractical on a large-scale.

In 2010, China produced about 500 tons of L-arabinose at the cost of more than 70 USD per kg. This prohibitive cost for obtaining high grade L-arabinose limits its use in applications such as food additives and drug synthesis. Therefore, there is a biotechnological and commercial interest in the development of new cost-effective and high-performance methods for obtaining high purity grade L-arabinose, which has biotechnological applications as a valuable starting material for manufacture of various life-saving drugs and other commercially important high-value products.

Xylose mother liquor is an acid hydrolysate by-product in the preparation of xylose from corncob or sugarcane bagasse and is rather abundant in China. It contains more than 350-400 g L^-1 ^xylose and 150-180 g L^-1 ^L-arabinose, as well as galactose and glucose. L-arabinose is difficult to separate and crystallize by general procedures due to the chemical complexity. Simulated moving bed chromatography has been practiced to separate L-arabinose from the mixture [9], but it demands expensive equipment and high running costs and has poor separation efficacy, limiting its large-scale use in industry. Therefore, development of a simpler method to obtain L-arabinose from xylose mother liquor is crucial.

In recent times, biological removal and bio-purification have become increasingly attractive approaches for producing high-value compounds from crude sugar feedstocks [10,11]. Most recently, Cheng *et al *designed a strategy to produce xylitol from xylose mother liquor through biological removal of growth inhibitors, glucose and L-arabinose, and enrich xylose which was then reduced to xylitol by a yeast [12]. Thus, a possible approach for the efficient separation of L-arabinose from xylose mother liquor would be to biologically deplete other sugars such as xylose, glucose and galactose from the xylose mother liquor, thus enriching L-arabinose in this liquor and facilitating its crystallization by simple methods.

In the present study, we identified *Pichia anomala *Y161 (China General Microbiological Culture Collection Center, accession no. 2480) that could grow well in a medium containing xylose, xylitol and galactose, but not L-arabinose, as a carbon source, and could also grow well in a medium containing 10-30% (v/v) xylose mother liquor. Therefore, this strain was used to biologically enrich L-arabinose from xylose mother liquor. Response surface methodology and Box-Wilson central composite design (CCD) were used to optimize the fermentation conditions by *P. anomala *Y161. Optimal fermentation conditions were deduced and were applied in 3 L fermentors to enrich L-arabinose by depleting xylose and glucose and some of the galactose present. Crystallized L-arabinose was obtained from this enriched fermentation medium by simple purification procedures.

## Methods

### Media and screening of yeast strains

To obtain a yeast strain that could utilize xylose, xylitol, galactose but not L-arabinose from xylose mother liquor, 306 yeast strains from our laboratory stock were screened by inoculating each culture in solid 1% (w/v) yeast nitrogen base medium (YNB, Difco, Detroit, MI, USA) containing 2% (w/v) agar, supplemented with either 1% (w/v) L-arabinose (YNA), 1% (w/v) xylose (YNX), 1% (w/v) xylitol (YNXL) or 1% (w/v) galactose (YNGL) and cultured at 28°C for 5 days. Cultures which could grow on other media but not on YNA were inoculated into solid YNB medium supplemented with 30% (v/v) xylose mother liquor (YNXM) and cultured at 28°C for 7 days. The culture that showed the fastest growth rate based on the colony size was selected. This strain was adapted to a medium containing 1% (w/v) yeast extract, 1% (w/v) tryptone, 30% (v/v) xylose mother liquor, 2% (w/v) agar (YPX) by repeated culturing for 30 cycles to further improve its adaptability in xylose mother liquor.

For taxonomic identification of this yeast strain, genomic DNA was extracted according to the simple phenol lysis method [13] to amplify partial sequences of its 18S rDNA and cytochrome c oxidase subunit 2 genes. The primers used for amplification of 18 S rDNA were: 5'-ATC CTG CCA GTA GTC ATA TGC TTG TCT C-3' and 5'-GAG GCC TCA CTA AGC CAT TCA ATC GGT A-3'; while those for the cytochrome c oxidase subunit 2 gene were: 5'-AATATAATGTTTTATTTAGTATTAATA-3' and 5'-TTTGATAGGCATCGCACTATGAGC-3'. PCR conditions were as follows: 95°C for 3 min, 30 cycles of denaturation at 94°C for 35 s, annealing at 61°C for 40 s, extension at 72°C for 90 s, and a final extension at 72°C for 10 min. Each PCR product was independently ligated into T-vector (pMD18, Takara, Dalian, China) and sequenced. Homology search was performed using the Basic local alignment tool (BLAST) available from National Center for Biotechnology Information (NCBI, Bethesda, MD, USA) [14].

### Yeast seed culture

Yeast pre-culture was prepared by transferring a loop of culture obtained as above to test tubes containing 10 mL seed culture medium (15% xylose mother liquor (v/v), 1% tryptone (w/v), 1% yeast extract (w/v)). The pre-culture was grown at 32°C on a rotary shaker at 200 rpm for 24 h and used to inoculate Erlenmeyer flasks (500 mL cap.) containing 90 mL of fermentation media with varying concentrations of xylose mother liquor (containing 153 g L^-1 ^L-arabinose, purchased from Jiahe Sugar Co. Ltd., Changyi City, Shandong Province, China). The cultural conditions were modified according to the experimental design. The speed of rotary shaker incubator was set to 250 rpm. All tests were conducted in duplicate and results presented are the mean values of two independent experiments.

### Enzymatic assays

Y161 culture was grown aerobically in 250 ml shaken flasks with 50 ml of medium containing 0.67% (w/v) YNB supplemented with 1% xylose (w/v), 1% xylitol (w/v), or 1% galactose (w/v), and incubated at 28°C on a rotary shaker at 250 rpm. 2 ml of cell suspension from each late-exponential phase culture (OD_600 _6-7) were harvested by centrifugation at 5000 *g *for 5 min, washed three times with extraction buffer (50 mM Tris-Cl [pH7.5], 0.1 mM EDTA and 2 mM dithiothreitol) and centrifuged at 5000 *g *for 5 min. The pelleted cells (about 35 mg dry weight cells per tube) were re-suspended in 400 μl of the above extraction buffer and were sonicated using an ultrasonic homogenizer in an ice water bath for fifty, 5 s bursts, interrupted by 5 s cooling intervals at 600 Watt. Crude cell-free extracts were obtained by recovering the supernatant after spinning down cell debris. This preparation was used to determine enzyme activities with L-arabinose, L-arabitol and L-xylulose as substrates and identify the enzymatic products by HPLC. For L-arabinose reductase (LAR, EC 1.1.1.21) and L-xylulose reductase (LXR, EC 1.1.1.10), activities were determined in Tris-HCl buffer (100 mM, pH 7.0), NADPH or NADH (0.2 mM) containing 1% (w/v, 66 mM) L-arabinose or 1% (v/v) L-xylulose. NAD- or NADP-dehydrogenase (EC 1.1.1.12) activities were assayed in glycine buffer (100 mM, pH 8.5), MgCl_2 _(50 mM), 1% (w/v, 66 mM) L-arabitol or 1% (w/v, 66 mM) xylitol containing either NAD or NADP (3.0 mM) respectively. All assays were carried out at 30°C for 5 min. One unit of enzyme activity was defined as the amount of activity necessary to convert 1 μmol of substrate to product (NADPH to NADP; or NADH to NAD; or NAD to NADH; or NADP to NADPH) per min. The protein concentration in crude extract was determined according to the Bradford method with bovine serum albumen (BSA) as a standard. The enzymatic reaction mixes (1 hour reaction) were extracted with phenol to remove proteins and 40 μL aliquots were applied to HPLC to identify the products.

### Experimental design and statistical analysis

Based on the results of our preliminary experiments, the selected yeast strain was grown under various cultural conditions to obtain optimized purity of L-arabinose. In these trials, three parameters were kept constant: medium contained 2.5% (w/v) yeast extract, pH was 5.5 and aeration was by shaking at 250 rpm. Yeast extract has been shown to be a good nitrogen source, increasing aldose (xylose) reductase activity, thus enhancing the metabolism rate of xylose [15]. An aeration rate of 250 rpm improved significantly the assimilation and metabolism rate of xylitol presumably by providing more cofactor NAD, thus shortening the fermentation time to enrich L-arabinose. Three parameters, namely, fermentation time (X1), temperature (X2) and concentration of xylose mother liquor in the medium (X3), were varied to obtain the optimized purity of L-arabinose according to the response surface methodology (RSM) (Design-Expert 7). A central composite design (CCD) was employed for fast multifactor screening to determine the most important independent factor [16]. A CCD at five levels was conducted with three independent variables of X1, X2 and X3 (Table 1). The complete design consisted of 20 experimental points (8 factorial points, 6 axial points and 6 center points) as shown in Table 2, and the experiments were carried out in a random order.

**Table 1:** Values of parameters used in the response surface design

Factors	Factor level				
	
	-2	-1	0	1	2
Fermentation time (h)	50	60	70	80	90

Fermentation temperature (°C)	30	31	32	33	34

Xylose mother liquor (%, v/v)	15	20	25	30	35

**Table 2:** Results of experimental trials (20 trials) conducted as per the central composite design (CCD) for optimization of yield in terms of purity of L-arabinose obtained from xylose mother liquor

Run No.	Time (h)	Temp. (°C)	Xylose mother liquid (%, v/v)	Purity degree of L-arabinose (%)
1	70	30	25	65.6
2	60	33	30	49.2
3	70	34	25	68.8
4	70	32	25	81.4
5	80	31	20	77.5
6	70	32	25	82.9
7	70	32	35	46.2
8	80	33	20	84.9
9	60	31	20	55.2
10	60	33	20	75.1
11	80	33	30	67.1
12	70	32	25	81.8
13	70	32	25	82.5
14	60	31	30	40.1
15	70	32	25	81.4
16	70	32	15	85.3
17	90	32	25	78.1
18	70	32	25	81.1
19	80	31	30	58.8
20	50	32	25	69.9

### Isolation and purification of L-arabinose

The yeast seed culture prepared as described above was inoculated at 5% inoculum size into four 3 L capacity fermentors (Bioflo 110, New Brunswick Scientific, Edison, NJ, USA) each containing 2 L of the optimum fermentation medium with 2.5% (w/v) yeast extract as nitrogen source at pH 5.5 and cultured under the optimum fermentation conditions obtained from response surface methodology. After fermentation, yeast cells were removed by centrifugation, and the supernatant was rotary decolorized with activated carbon at 80°C. Then the colorless fermentation broth thus obtained was clarified by removal of activated carbon via filtration, and was applied to ion-exchange resin columns for removal of metal ions, which was verified by a conductivity less than 20 μs/cm. The solution was adjusted to contain 70% (w/v) of L-arabinose by concentrating, and cooled gradually from 70°C to 4°C to allow L-arabinose crystallized. White powdery crystals precipitated from solution, then were centrifuged and washed with 95% ethanol, and dried. The purity of L-arabinose was estimated by HPLC.

### Analytical methods

L-arabinose, xylose, glucose, galactose and xylitol were estimated by resolving samples on prepacked analytical HPLC columns (Shodex SPO 810, 8 × 30 mm, Pb^2+ ^cation exchange column), with distilled water as eluent, at a flow rate of 1.0 mL/min, at 70°C. Sugars were identified using a refractive index detector (Shodex RI 101).

## Results and discussion

### Screening, selection and identification of yeast *Pichia anomala *Y161

**Figure 1:**
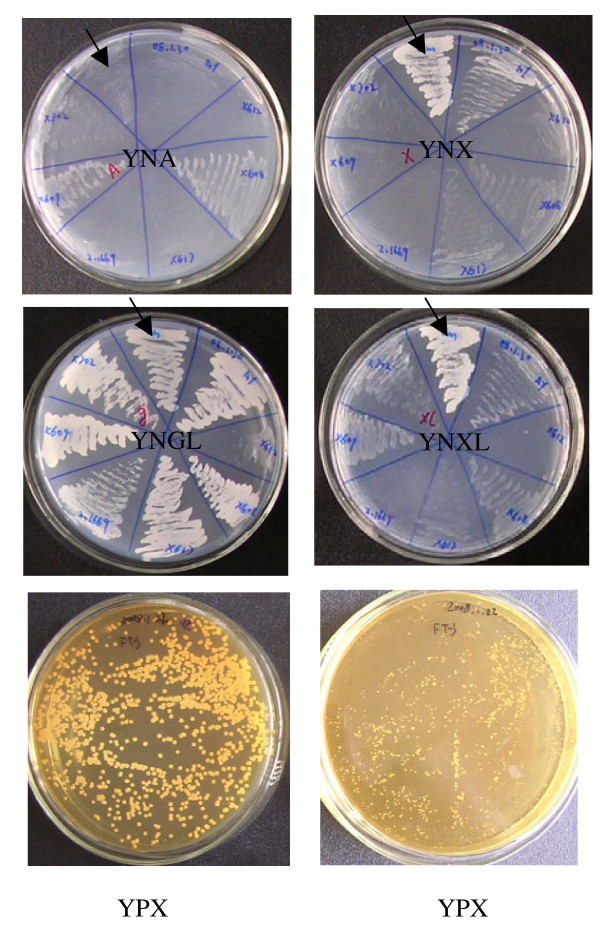
**Growth of yeast on solid medium containing different sugars**. Screening of 306 yeast strains was performed by observing their growth on YNB medium supplemented with either L-arabinose (YNA), xylose (YNX), xylitol (YNXL) or xylose mother liquor (YPX). Black arrows indicate the sector inoculated with the selected strain that was capable of growth on all the above medium except YNA. After repeated culturing of this strain in YPX medium for 30 cycles, it appeared to have adapted as seen from the comparison of its growth (bottom left panel) with an unadapted culture (bottom right panel). The adapted strain was then designated as strain Y161. On YNA medium, Y161 showed poor growth in the first 3 days, and no further increase in colony size or number was observed in the next 4 days.

Among 306 strains of yeasts that were screened on YNB-based media supplemented with different sugars, all strains could utilize galactose as they were capable of growth on solid YNGL medium. Of these, four strains grew well on solid YNX, YNXL medium but grew poorly on YNA. Cultures of these four yeast strains were diluted appropriately with sterilized water and spread onto solid YNXM medium as described in Materials and Methods. Based on the relative colony sizes, one strain was found to grow faster than the other three and was selected for further experiments. After 30 successive cycles of adaptation to YPX medium containing 30% (v/v) xylose mother liquor, the culture of this strain was found to grow faster (colony size 1.0-1.2 mm) than an unadapted culture (colony size 0.2-0.4 mm) on YPX medium after 5 days at 28°C. This yeast strain which utilizes well xylose, xylitol, galactose but not L-arabinose, grew fastest on YNXM and was adapted to YPX medium was designated as Y161. The growth characteristics of yeast Y161 on YNA, YNX, YNXL, YNGL and YPX solid medium are shown in Figure 1.

The xylose mother liquor contains more than 350-400 g L^-1 ^xylose and 150-200 g L^-1 ^galactose plus glucose in addition to 150-180 g L^-1 ^L-arabinose. The approach followed here for enriching L-arabinose was based on elimination of other sugars from the xylose mother liquor. Hence the screen applied was aimed at selecting a yeast strain with excellent properties for transport and rapid metabolism of glucose, galactose and xylose, and capacity for adaptation to high concentration of xylose mother liquor. The yeast strain Y161 met these requirements and was adapted to a medium containing 30% (v/v) xylose mother liquor.

The taxonomic identity of Y161 was established based on cloning and sequencing of its 18S rDNA and cytochrome c oxidase subunit 2 genes (see Methods). The two nucleotide sequences were analyzed for homology by the BLAST search available from NCBI. The results of this analysis showed that the 1.7 kb 18S rDNA fragment from yeast Y161 (GenBank accession No. HQ901201) had 99% identity with that from *Pichia anomala *strain M34-1, strain HS054 and strain IFO 10213^T ^[17], as well as with that from *Pichia subpelliculosa *NRRL Y-1683, *Pichia sydowiorum *NRRL Y-7130, *Pichia ciferrii *NRRL Y-1031, *Candida silvicultrix *NRRL Y-7789 [18]. Similarly, the 580 bp fragment of cytochrome c oxidase subunit 2 gene from yeast Y161 (GenBank accession No. HQ901202) showed 100% identity with a homologous sequence from *Pichia anomala *isolate WM 825 [19] and 99% identity to that from *Wickerhamomyces anomalus *NRRL Y-366 [20]. Both the sequenced DNA fragments showed less than 90% identity with corresponding sequences from other yeast strains. Thus, the yeast Y161 was identified as *Pichia anomala*.

### Growth pattern of Y161 in YPX liquid medium

**Figure 2:**
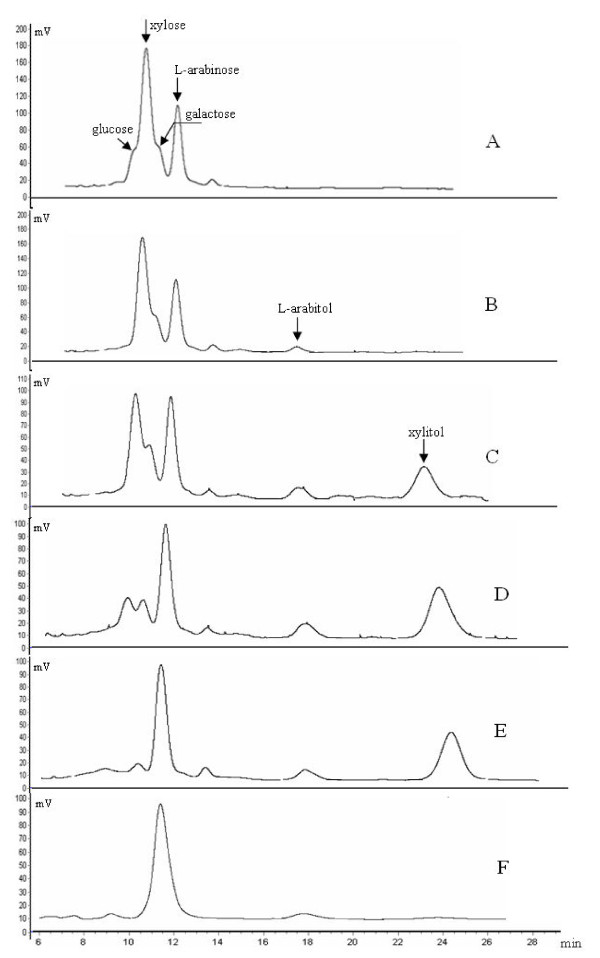
**Sugar assimilation patterns during growth of Y161 in YPX medium containing 10% (v/v) xylose mother liquor**. Aliquots were removed at different times during fermentation (A, 0 h; B, 8 h; C, 20 h; D, 30 h; E, 36 h; F, 48 h) and analyzed by HPLC to identify the residual sugars as indicated.

Y161 cells were grown in 500 ml capacity flasks containing 50 ml YPX liquid medium supplemented with 10% (v/v) xylose mother liquor and incubated at 32°C on an orbital shaker at 250 rpm. Samples were withdrawn periodically to estimate the content change of glucose, xylose, L-arabinose, galactose, L-arabitol and xylitol during cultivation (Figure 2). The xylose mother liquor used in this study contained glucose, xylose, galactose, L-arabinose and an unknown substance (Figure 2A). The amount of xylose was approximately 2.5 fold higher than that of L-arabinose. Glucose was the first sugar to be metabolized by Y161, while trace amounts of L-arabitol were produced after 8 h indicating a low level reduction of arabinose (Figure 2B). After 20 h fermentation, the amount of xylose decreased significantly and was almost equal to that of L-arabinose. The xylose appeared to be first reduced to xylitol, of which 50% was secreted into the external medium (Figure 2C). After 30 h of fermentation, the amount of xylose and galactose decreased to 3.2 g L^-1 ^and 2.5 g L^-1^, and the amount of xylitol increased to 12.3 g L^-1^. Presumably, xylose was metabolized and transformed to biomass and energy, while L-arabitol was still unutilized (Figure 2D). After 36 h, xylose from the medium had reached undetectable levels and the amount of xylitol started to decrease. Trace amounts of galactose and L-arabitol were found in medium (Figure 2E). After 48 h, xylitol was exhausted as it was almost undetectable, while trace amounts of L-arabitol remained in the medium (less than 1.5 g L^-1^). At this point (48 h), the concentration of L-arabinose was 13.5 g L^-1^. Comparing to the starting fermentation medium which contains 15.3 g L^-1 ^L-arabinose, 88.2% yield of L-arabinose was achieved in the final fermentation medium. Only 1.8 g L^-1 ^L-arabinose was reduced to L-arabitol, indicating that yeast Y161 could not utilize well L-arabinose and showed a weak ability to reduce L-arabinose to L-arabitol (Figure 2F). After 48 h of fermentation, the degree of purity of L-arabinose increased more than 3-fold, from the initial 26.2% to 85.6% in terms of total sugars.

Xylose is a major constituent of plant material such as corn cob and sugarcane bagasse. Xylose metabolic pathway is therefore well developed in microorganisms that live on decaying plant materials, including most strains of yeasts such as *Pichia stiptis *[21], *Candida tropicalis *[22], *Candida shehatae *[23] and *H. anomala *used in this study. The metabolism of xylose in yeasts has been described in detail in the literature [24-27]. Xylose transport across the plasma membrane is the first step in xylose metabolism pathway in yeasts. It is known that xylose is taken up by active xylose/proton symporter that mostly appeared to be associated with glucose transport and showed a much higher affinity for glucose than for xylose [28]. The uptake of xylose is highly influenced by the presence of glucose. During the fermentation of xylose mother liquor by the yeast Y161 in this study, xylose uptake was not observed until glucose was exhausted and xylitol was detected in medium only after 8 h (Figure 2B), indicating that glucose has a strong inhibitory effect on xylose uptake. Moreover, glucose was shown to be an inhibitor of the intracellular enzymes responsible for the catabolism of xylose, for example reduction to xylitol [29]. Once glucose has been consumed, enzymes for xylose metabolism are activated and xylitol accumulates intracellularly and is then secreted to the medium (Figure 2C-E). Extracellular xylitol concentration reached a maximum value just before nearly all of the xylose was consumed (Figure 2D). The xylitol appears to have been utilized for generating biomass and energy during the later phases of fermentation, as seen from the subsequent decline in concentration of extracellular xylitol until it was undetectable (Figure 2E-F). Ultimately, L-arabinose was the predominant sugar left in the fermentation medium, thus reaching a high degree of purity.

Similar to xylose, L-arabinose is also a natural sugar and a component of plant carbohydrates which is commonly found in hemicellulose such as L-arabinnans, L-arabino-xylans in corn fiber which is associated to xylose. Approximately a third of L-arabino-xylans in corn fiber is L-arabinose. Many types of yeast harbor the L-arabinose metabolism pathway as well as the xylose metabolism pathway. Among these, *Candida arabinofermentans *PYCC 5603^T ^and *Pichia guilliermondii *PYCC 3012 received most attentions [28,30,31]. L-arabinose transport across the yeast outer membrane is the first step in its metabolism. In yeast *C. arabinofermentans *PYCC 5603^T ^and *P. guilliermondii *PYCC 3012 which have a high L-arabinose uptake rate, there are two L-arabinose transport systems, one is the L-arabinose/proton symporter with high-affinity transport components, and the other is a facilitated diffusion transport system with a relative low-affinity component. Once inside the cytoplasm, L-arabinose is reduced to L-arabitol by an unspecific NADPH-linked aldose reductase which could also reduce xylose to xylitol [10,28]. L-arabitol is then oxidized to L-xylulose by an NAD-dependent L-arabitol 4-dehydrogenase (LAD), and L-xylulose was reduced to xylitol, a common intermediate to the xylose metabolism pathway. Y161 could efficiently metabolize xylose and xylitol but showed a poor ability to metabolize L-arabinose, probably because of the low activities of the enzymes, L-arabinose reductase or L-arabitol 4-dehydrogenase. Only a small amount of L-arabitol (less than 1.2 g/L) was produced when Y161 was cultured in YPX medium containing 10% xylose mother liquor (Figure 2B-E). Subsequently, in the later stages of fermentation, like in the case of xylitol, the amount of extracellular L-arabitol decreased (Figure 2F). This indicates that a certain amount of L-arabinose could be transported across the plasma membrane and was metabolized. However, Y161 grew poorly, if at all, on YNA agar/liquid medium, indicating that despite a detectable level of transport of this sugar, further metabolism may not be efficient due to low activities of enzymes involved in L-arabinose metabolism, such as L-arabinose specific reductase (LAR), LAD or L-xylulose reductase (LXR).

### Enzymatic activities and products

In order to investigate the possible reasons for poor utilization of L-arabinose by Y161, activities of four known enzymes, namely, LAR, LAD LXR and XDH, involved in L-arabinose metabolism were assayed, in crude cell-free extracts, in the presence of appropriate cofactors (Table 3). In extracts of Y161 cultures grown on galactose as a carbon source the activity of LAR was 0.032 U mg^-1 ^using NADPH as cofactor, and decreased to a tenth with NADH as cofactor, indicating a preference for NADPH. LXR activity was strictly NADPH-dependent. Weak LAD activity of ~0.003 U mg^-1 ^was found in these galactose-grown cells, which increased to around 0.012 and 0.018 U mg^-1 ^when the cells were grown on xylose and xylitol, respectively, suggesting LAD activity is less effectively induced by xylose or xylitol. Similarly, LAR and LXR were also less effectively induced by xylose or xylitol. In contrast, XDH showed both, NADP- and NAD-dependent activity, with a higher preference for NAD. An XDH activity of ~0.86 U mg^-1 ^was found in galactose-grown cells, which increased to 5.0 and 5.2 U mg^-1 ^when the cells were grown on xylose or xylitol, respectively. This suggests that XDH activity is more effectively induced by xylose or xylitol compared with the other three enzymes tested, which might explain why yeast Y161 utilizes xylose or xylitol efficiently (Figure 1). In contrast, the very low LAR, LAD and LXR activities inY161 might explain its poor ability to utilize L-arabinose (Figure 1).

**Table 3:** Specific activities of L-arabinose reductase (LAR), L-arabitol 4-dehydrogenase (LAD), L-xylulose reductase (LXR) and xylitol dehydrogenase (XDH) in crude cell free extracts of Y161 grown on different carbon sources

**Specific activity (U, mg of protein**^**-1**^**)**				
		
Enzyme	Cofactors	Grown on galactose	Grown on xylose	Grown on xylitol
LAR	NADPH	0.032	0.16	0.12
	NADH	0.002	0.014	0.011
LAD	NAD	0.003	0.012	0.018
	NADP	ND	ND	ND
LXR	NADPH	0.004	0.021	0.024
	NADH	ND	ND	ND
XDH	NAD	0.86	5.0	5.2
	NADP	0.007	0.18	0.26

Values presented are means of duplicates

ND, not detectable.

**Figure 3:**
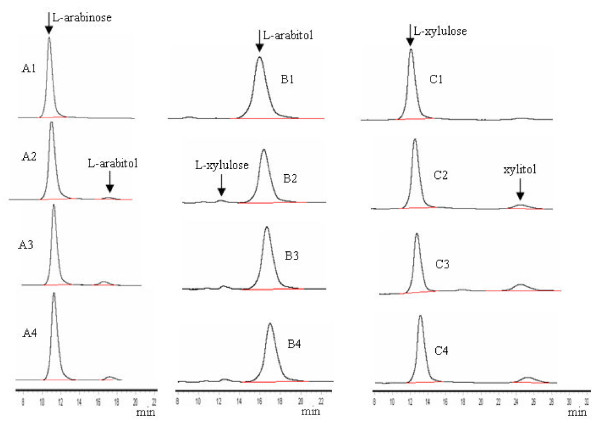
**HPLC analysis of enzymatic reaction products**. Crude cell free extracts were used for analysis of various enzyme activities. The reactions were allowed to run overnight and the solutions were extracted with phenol to remove proteins. Aliquots (40 μl) from the various reactions were applied to HPLC column: A1, 1% (w/v) L-arabinose standard, no enzymatic reaction; A2-A4, 1% L-arabinose plus crude extracts of Y161 grown with galactose, xylose or xylitol as a carbon source, respectively. B1, 1% (w/v) L-arabitol standard, no enzyme; B2-B4, L-arabitol plus crude extracts of Y161 grown with galactose, xylose or xylitol as a carbon source, respectively. C1, 1% (v/v) L-xylulose standard; C2-C4, L-xylulose plus crude extracts of Y161 grown with galactose, xylose or xylitol as a carbon source, respectively.

Through HPLC analysis, we also identified the enzymatic reaction products with L-arabinose, L-arabitol and L-xylulose as substrates and NADPH or NAD as cofactors. These reactions were performed with crude cell free extracts of Y161 grown on medium containing galactose, xylose or xylitol as a carbon source. L-arabinose was reduced to L-arabitol by the above crude extracts of Y161 in the presence of NADPH as a cofactor (Figure 3 A2-A4). L-xylulose was formed with L-arabitol as substrate and NAD as cofactor, using the crude extracts of Y161 (Figure 3 B2-B4) while L-xylulose was the reduced to xylitol (Figure 3 C2-C4). It should be noted that the amount of L-xylulose oxidized from L-arabitol was significantly less than the amount of L-arabitol reduced from L-arabinose or xylitol reduced from L-xylulose (Figure 3), indicating that the activity of dehydrogenase harboring LAD was very low, consistent with the results shown in Table 3. Y161 did not grow in a medium with L-arabitol as a carbon source (data not shown), although the cells exhibited low LAD activity. There are a number of instances of a polyol oxidized by the cell-free crude extracts of a yeast, but not utilized for growth. *Candida utilis *could not utilize D-glucitol but it could be oxidized in crude extract, due to exogenous D-glucitol, which does not enter intact cells of this strain [32].

### Experimental design

In general, the cost-effectiveness of any fermentation process is governed by several parameters, including the cost of the raw material and the efficacy of its conversion into the desired product; the cost of energy consumption, influenced by the time required to operate the fermentor, and the optimum temperature to provide the maximum yield. Here, we modeled optimization of the fermentation process based on three parameters, namely, time (X1), temperature (X2) and concentration of xylose mother liquor in the medium (X3). These three variables were tested in a total of 20 trial runs in the current CCD as depicted in the experimental design and the results were obtained from experiments (Table 2). Maximum purity of L-arabinose (85.3%) was achieved under the experimental conditions of fermentation time 70 h, fermentation temperature 32°C in a medium containing 15% (v/v) xylose mother liquor. By applying multiple regression analysis on the experimental data, the response variable, Y, for purity of L-arabinose and the three test variables were related with the second-order polynomial equation (using the above codes) as given below:

The statistical significance of the regression model was checked by *F*-test. The fit of the polynomial model (R^2^) was calculated to be 0.8345, indicating that 83.45% of the variability in the response could be explained by the model. Generally, a regression model having an R^2 ^value higher than 0.8 is considered to have a high correlation [33]. Predicted R-squared was -0.3302, implying that the overall mean is a better predictor of response than the current model. The response equation represents a suitable model for L-arabinose enrichment from xylose mother liquor.

**Figure 4:**
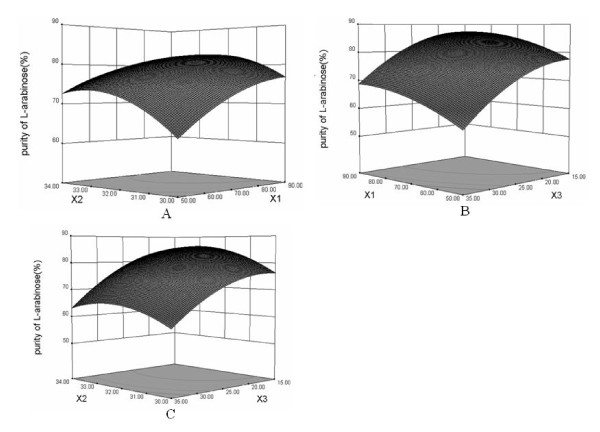
**Response surface for the enrichment of L-arabinose by *Pichia anomala *Y161**. Impact on the purity of L-arabinose (%) of the interactions between the different fermentation parameters was estimated: A: fermentation time (X1, h) and fermentation temperature (X2, °C); B: fermentation time (X1, h) and concentration of xylose mother liquor in the medium (X3, %, v/v); C: fermentation temperature (X2, °C) and the concentration of xylose mother liquor (X3, %, v/v)

3-D response surfaces plots were employed to determine the interaction of the fermentation conditions and the optimum levels of the parameters that have a significant impact on the purity of L-arabinose. In Figure 4A, when the 3-D response plot was developed for the enrichment of L-arabinose with varying fermentation time (X1) and fermentation temperature (X2) at a set concentration of xylose mother liquor (X3, 25%), the degree of purity of L-arabinose increased with increasing fermentation time, and was enhanced with increasing fermentation temperature from 31 to 32.5°C, but then dropped at temperatures between 32.5°C and 34°C, indicating that the fermentation temperature has a crucial influence on the outcome.

In Figure 4B, the fermentation temperature was fixed at the 0 level (32°C). The purity degree of L-arabinose increased when the concentration of the xylose mother liquor was reduced from 35% to 15%. The degree of purity of L-arabinose increased with the prolonged fermentation and reached the maximum value at 85 h. Figure 4C shows the 3-D response surface diagram and the contour diagram with varying ratios of xylose mother liquor and fermentation temperature at a fixed fermentation time (0 level, 70 h). The concentration of xylose mother liquor had a negative effect on the degree of purity of L-arabinose. The latter increased with decreasing concentration of xylose mother liquor.

Among the three fermentation parameters studied, concentration of xylose mother liquor in the medium had the highest impact on the degree of purity of L-arabinose, followed by fermentation time and fermentation temperature, based on the regression coefficients in the quadratic polynomial model (Table 4).

**Table 4:** Analysis of variance of the degree of purity of L-arabinose calculated with a quadratic equation obtained from experimental results

Source	Sum of Squares	df	Mean Square	*F*-Value	Probability > F
Model	3093.963	9	343.7737	5.603423	0.0063
X1	452.6256	1	452.6256	7.377682	0.0217
X2	163.2006	1	163.2006	2.660129	0.1339
X3	1515.156	1	1515.156	24.69665	0.0006
(X1)(X2)	22.11125	1	22.11125	0.360408	0.5616
(X1)(X3)	2.53125	1	2.53125	0.041259	0.8431
(X2)(X3)	12.25125	1	12.25125	0.199692	0.6645
(X1)^2^	190.7719	1	190.7719	3.109534	0.1083
(X2)^2^	498.9091	1	498.9091	8.132091	0.0172
(X3)^2^	583.413	1	583.413	9.509483	0.0116
Residual	613.5065	10	61.35065		
Lack of Fit	611.0115	5	122.2023	244.8944	< 0.0001
Pure Error	2.495	5	0.499		
Total	3707.47	19			

X1, X2 and X3 correspond to fermentation time, temperature and concentration of xylose mother liquor, respectively; df, degrees of freedom

To verify the predicted results of the model for optimal purity of L-arabinose, experiments were performed in duplicate under the optimized fermentation conditions: medium volume 100 mL, inoculation volume 5% (v/v), fermentation time 75 h, fermentation temperature 32.5°C, 21% (v/v) xylose mother liquor, 2.5% yeast extract and pH 5.5 in 500 mL Erlenmeyer flask. The degree of purity of L-arabinose obtained from xylose mother liquor reached 86.1% by HPLC analysis (data not shown). The predicted value 87.05% is in good agreement with experimental values, proving the accuracy of the model and the existence of optimum points.

### Purification of L-arabinose from fermentation medium

The purity of L-arabinose in the fermentation medium using Y161 could reach around 86% of total sugars, which exceeds the purity requirement for crystallization of L-arabinose from a solution. From 8 L fermentation broth of Y161containing 1.6 L xylose mother liquor, 165 g white powdery crystals of L-arabinose was obtained, an yield of 103.1 g L-arabinose per L of xylose mother liquor. Theoretically, 240 g L-arabinose should be obtained from the 8 L fermentation medium containing 1.6 L xylose mother liquor (153 g L-arabinose/L). About 30 g L-arabinose was lost during the process of purification and there is a residual of 45 g L-arabinose in the fermentation medium which could not be recovered due to the increased conductivity and impurities which interfere with the L-arabinose crystallization. The final preparation of the purified white powder was dissolved in water and applied for HPLC analysis to confirm a purity of 99% (data not shown).

Recently, yeast strains, especially *S.cerevisiae*, have received intensive studies to act as cell factories in green chemistry. Many natural products used in pharmaceutical and nutraceutical industries such as isoprenoids, flavonoids, and other fine chemicals could be efficiently synthesized using *S.cerevisiae *as the green synthesis factory [34,35]. On the other hand, yeast cells could also be used as green decomposition factory to biodegrade or metabolize unwanted compounds to enrich the desired product [36,37]. Here, the yeast strain *P. anomala *Y161 was employed as a green decomposition factory to metabolize other low-valued sugars and enrich high-valued L-arabinose from compositionally complex xylose mother liquor. To our knowledge, this is the first attempt to apply this biotechnological method for the preparation of L-arabinose from xylose mother liquor. Latter is a viscous and compositionally complex, low-value by-product of the xylose production industry. Further studies on the feasibility of this novel strategy for the scaled-up preparation of L-arabinose from xylose mother liquor are currently underway.

## Conclusion

In this study, we described a novel biological method to obtain high purity L-arabinose from xylose mother liquor. A strain of *P. anomala *Y161 was recovered in a screen designed to identify a yeast strain with a poor capacity of utilizing L-arabinose but a good capacity for utilizing other sugars that are present in xylose mother liquor. Optimum fermentation conditions were deduced by evaluating the effect of three independent variables using RSM. Under the optimized conditions, a high degree of purity of L-arabinose (86.1%) was obtained, and white powdery L-arabinose could be crystallized from this fermentation medium by following standard, simple techniques. The method described here appears to be amenable for large scale biotechnological applications.

## Competing interests

The authors declare that they have no competing interests.

## Authors' contributions

CHR and JMG designed research; CHR and WHH performed the yeast strain screening and molecular identification; LJY performed HPLC analysis and fermentation experiments; LSJ and DZX provided advice on organizing the manuscript and on editorial quality; CHR performed the literature review and drafted the manuscript. All authors have read and approved the final version of the manuscript.
